# Human Infection with Avian Influenza Virus, Pakistan, 2007

**DOI:** 10.3201/eid1706.091652

**Published:** 2011-06

**Authors:** Mukhtiar Zaman, Saadia Ashraf, Nancy A. Dreyer, Stephen Toovey

**Affiliations:** Author affiliations: Khyber Teaching Hospital, Peshawar, Pakistan (M. Zaman, S. Ashraf);; Outcome Sciences, Cambridge, Massachusetts, USA (N.A. Dreyer);; University College and Royal Free Medical School, London, UK (S. Toovey)

**Keywords:** H5N1, avian influenza, influenza A, influenza, Pakistan, zoonosis, nosocomial, viruses, dispatch

## Abstract

Human infection with avian influenza (H5N1) virus raises concern for the possibility of a pandemic. We report 20 cases, which ranged from asymptomatic to fatal, in Pakistan in 2007. These cases indicate human-to-human-to-human transmission of this virus, and the number of cases may be higher than realized.

Evidence of human-to-human transmission of influenza A (H5N1) virus raises concern over a possible pandemic (*1*). Previous epidemiologic investigation of the outbreak of influenza (H5N1) among persons in the Northwest Frontier Province of Pakistan (Figure 1) in 2007 found 5 cases—3 confirmed, 1 asymptomatic, and 1 probable—as defined by the World Health Organization (WHO) (*2*). We report a larger set of 20 cases during this outbreak in Pakistan, supporting human-to-human-to-human transmission.

**Figure 1:**
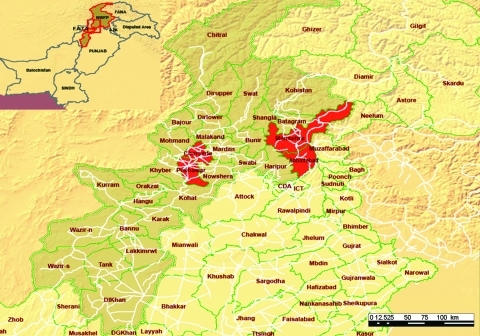
Areas of influenza (H5N1) cases in humans, Pakistan, 2007. Red shading indicates districts that reported suspected human cases of influenza (H5N1). Light brown shading indicates Northwest Frontier Province. Source: World Health Organization (WHO). Districts of avian influenza suspected cases in Northwest Frontier Province, Pakistan. WHO map no. WHO-PAK-002 (www.whopak.org/disaster).

## The Cases

Records were examined from all hospitals that treated patients with influenza (H5N1) virus in Northwest Frontier Province during 2007. Data were rendered anonymous and entered into a secure database with predetermined clinical and epidemiologic fields. Cases matching predefined criteria (Table 1) were classified as laboratory confirmed, likely, or possible. Cases not meeting classification criteria were excluded. We slightly modified WHO criteria to resemble criteria that clinicians might adopt during an actual outbreak, especially in a resource-poor setting (*3*).

**Table 1:** Case classification definitions used to diagnose influenza (H5N1) infection in humans, Pakistan, 2007*

Classification	Definition
Laboratory confirmed	Laboratory confirmation of influenza (H5N1) virus at local/regional or World Health Organization confirmatory laboratory
Likely	
Definition 1	Epidemiologically linked by time, place, and exposure to a likely or confirmed human or avian influenza (H5N1) case AND
	Equivocal test OR positive laboratory confirmation of an influenza A virus infection but insufficient laboratory evidence for influenza (H5N1) virus infection AND
	Clinical signs or symptoms consistent with disease (regardless of severity): fever or flu-like
Definition 2	Epidemiologically linked by time, place, and exposure to a likely or confirmed influenza (H5N1) case-patient AND
	Death due to unexplained acute respiratory illness AND
	Negative test/test not performed
Possible	Epidemiologically linked by time, place and exposure to a likely or confirmed human or avian influenza (H5N1) case AND
	Test not performed/negative test AND
	Clinical signs and symptoms consistent with disease (regardless of severity): fever or flu-like
Noncase	Confirmed positive for non-H5N1 influenza A virus subtype OR
	Patient condition determined to have etiology other than avian influenza

We identified 20 cases—4 laboratory confirmed, 7 likely, and 9 possible—resulting in a ratio of 4 likely/possible cases for each laboratory-confirmed case. Median age was 29 years (range 7–60 years) for all patients and 30 years (range 23–35 years) for confirmed case-patients; 16 (80%) patients were male. The infecting exposure could not be established for all patients because multiple exposures, human and avian, were recorded for some. Of the 4 patients with laboratory-confirmed cases, 3 were treated with oseltamivir (2 [67%] of whom survived), and 1 had asymptomatic disease and received no antiviral treatment.

Signs and symptoms were mainly those of a febrile influenza-like illness (Table 2), although 1 patient with a laboratory-confirmed case was asymptomatic (microneutralization titer 320, Western blot positive, throat swab positive for H5 by reverse transcription–PCR); this case-patient was also described in a previous epidemiologic investigation (*2*). Gastrointestinal signs and symptoms were not prominent, and neurologic signs were not reported.

**Table 2:** Clinical characteristics for persons with reported cases of influenza (H5N1), Pakistan, 2007

Clinical sign or symptom	Diagnostic certainty, no./total (%)		
	Laboratory confirmed	Likely	Possible
Respiratory			
Abnormal breath sounds (wheezing, rales, stridor, rhonchi)	1/2 (50)	1/5 (20)	2/5 (40)
Excessive sputum production	0/1 (0)	0/4 (0)	2/6 (33)
Rhinorrhea/nasal discharge	1/2 (50)	1/4 (25)	1/5 (20)
Unexplained respiratory illness with cough, shortness of breath, or difficulty breathing	1/3 (33)	4/7 (57)	7/9 (78)
Sore throat/pharyngitis	1/1 (100)	0/4 (0)	3/6 (50)
Tachypnea	0/2 (0)	1/5 (20)	1/5 (20)
Cyanosis	0/1 (0)	0/4 (0)	1/3 (33)
Chest pain	2/2 (100)	1/1 (100)	3/5 (60)
Pleural effusion	0	0	1/3 (33)
Hemoptysis	0	0	1/1 (100)
Orthopnea	0	0	0 (0)
Gastrointestinal			
Diarrhea	0/1 (0)	1/4 (25)	2/5 (40)
Abdominal pain	0	0	0
Vomiting	0/4 (0)	0/7 (0)	1/9 (11)
Rectal bleeding	0	0	0/1 (0)
Other			
Fever	3/4 (75)	7/7 (100)	6/9 (89)
Headache	2/3 (67)	2/4 (50)	2/5 (40)
Body aches	1/1 (100)	0	1/1 (100)
Backache	0	0	1/1 (100)
Pericardial effusion	0	0	0/1 (0)
Nonpitting pedal edema	0	0	0/1 (0)
Fatigue or malaise	0/4 (75)	0/7 (0)	1/9 (11)
Myalgia	0/1 (0)	1/3 (33)	2/4 (50)
Tachycardia	0	1/1 (100)	0

**Figure 2:**
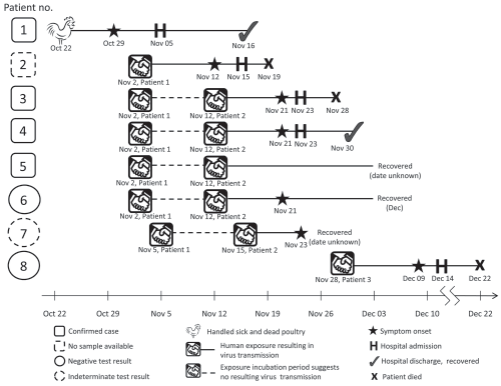
Path of infection of influenza (H5N1), Pakistan, 2007. During October 22–30, patient 1 worked culling infected chickens; on November 2, he moved home and had contact with 4 brothers (patients 2–5) and possibly a cousin (patient 6). He was hospitalized on November 5 and transferred to an intensive care unit the next day. His cousin cared for him and became patient 6; his attending doctor became patient 7. On November 23, patient 3 was hospitalized and on November 28 was transferred to an intensive care unit; during this time, patient 8 frequently visited his wife in the same intensive care unit.

The first 8 cases constituted a cluster (Figure 2). The index case-patient (patient 1) had culled influenza (H5N1) virus–infected poultry. After becoming febrile (38°C) while in Abbottabad, he traveled by public transportation to his family home in Peshawar. His illness progressed and on November 5, 2007, he was admitted to Khyber Teaching Hospital, where the diagnosis of influenza (H5N1) infection was made. Infection appeared to spread initially from household family contacts (patients 2–6) to medical staff (patient 7, who had positive PCR but negative microneutralization test results) and to a frequent visitor to the intensive care unit (patient 8).

As previously noted (*2*), the extended period from the time persons were exposed to the index case-patient, during which family members became ill, points to human-to-human-to-human transmission; patient 2 probably accounted for intermediary or second-generation infection. The chain of infection illustrated in Figure 2 suggests that further human-to-human-to-human transmission might have occurred and suggests nosocomial transmission. Of note, patient 6 (a cousin of the index case-patient) had a microneutralization titer of 80 but a negative Western blot result. Although 4 contacts of patient 6 exhibited no signs or symptoms of influenza, they did have positive H5 microneutralization titers ranging from 80 to 160.

No evidence epidemiologically links the remaining 12 patients to the 8 patients in the cluster; each of the 12 either had direct contact with influenza (H5N1) virus–infected poultry or was near healthy or diseased poultry before symptom onset. Three patients worked on poultry farms: 1 had taken a sample from an influenza (H5N1) virus–infected chicken, 1 was directly involved in culling, and 1 was indirectly exposed to live poultry. Eight patients had negative test results for influenza (H5N1) virus, and 3 had positive results from the National Institute of Health Islamabad but negative confirmatory-testing results from WHO; 1 patient died before samples could be taken. Different laboratories reported conflicting results with respect to confirmation of infection, possibly because of the difficulties of complying with specimen-handling requirements in resource-poor settings. Clinical details of these cases are shown in Table 2.

## Conclusions

The preponderance of male patients is probably explained by sociocultural factors; the index case-patient was a poultry culler, a male-dominated task, and shared accommodation with male family members. Health care–seeking behavior may also account for this finding.

The human-to-human transmission from the index case-patient to at least some household contacts seems clear, and the extended period over which these contacts became ill supports subsequent human-to-human transmission. Figure 2 supports the conclusion that patient 2 initiated a chain of infection in which further human-to-human transmission to patients 7 and 8 occurred. Possible nosocomial transmission is of concern because full implementation of isolation procedures in resource-poor settings may be problematic.

Although virologically supported probable human-to-human transmission of influenza (H5N1) virus has been documented, it has been thought to occur only with prolonged and close contact (*4*). Household clustering and the difficulty of establishing exact virus exposures have encumbered efforts to investigate possible human-to-human transmission (*5*). Modeling has (*6*) suggested human-to-human transmission in Indonesia, but the utility of statistical modeling unsupported by field data has been questioned (*7*).

Although the index case-patient traveled by public transportation from Abbottabad, where he acquired his infection, no infections were reported for anyone other than household contacts, who were all related and exposed at his family home at Peshawar. In contrast, patients 2 and 6 might have spread infection through less intimate contact, which raises 2 questions. Might some persons shed virus more efficiently than others, possibly in greater quantity? And what role might host factors play in susceptibility to influenza (H5N1) virus infection and disease? A degree of virus adaptation to humans might also have occurred, although absence of sustained community transmission argues against this possibility.

Of concern is the 4:1 ratio of likely/possible to laboratory-confirmed cases, suggesting that official tallies understate true incidence of infection. Factors that may contribute to undercounting are the difficulty of obtaining virologic confirmation or of storing and transporting samples in resource-poor settings and reluctance by relatives to consent to autopsy. Another reason to believe that less fulminant cases may go unreported is the occurrence in Pakistan, and elsewhere, of clinically mild and asymptomatic cases (*5**,**8**–**14*), indicating that influenza (H5N1) virus may cause a spectrum of illness. The demonstration during the 1997 Hong Kong outbreak of influenza (H5N1) with seroconversion in apparently asymptomatic health care workers and social contacts suggests human-to-human transmission, although in Hanoi, no transmission to health care workers was detected (*8**,**13**,**15*). Also contributing to underreporting are the predominant clinical signs of undifferentiated influenza-like illness observed in Pakistan and elsewhere, which, unless clinical deterioration occurred, would be unremarkable in many tropical settings. Although the survival rate was greater for patients who received oseltamivir, the small number of patients and the inclusion of those with mild and asymptomatic illness prevent meaningful statistical comparison.

Several features of the outbreak are unusual or give cause for concern: human-to-human-to-human transmission, possible nosocomial transmission, occurrence of mild and asymptomatic cases, and difficulties of establishing laboratory confirmation of likely and possible cases (which also prevented genotypic matching of specimens from primary and putative secondary cases). Taken together, these features suggest that current surveillance might undercount the extent of human infection with influenza (H5N1) virus and that human-to-human transmission might possibly be associated with less severe disease.
